# Supplementary material for: Whole genome sequencing of Borrelia burgdorferi isolates reveals linked clusters of plasmid-borne accessory genome elements associated with virulence
Source: bioRxiv. 2023 Feb 27:2023.02.26.530159. Preprint. [Version 1] doi: 10.1101/2023.02.26.530159 (PMC10002713; doi:10.1101/2023.02.26.530159)
Supplement: Supplement 1 [file NIHPP2023.02.26.530159v1-supplement-1.pdf]

bioRxiv preprint doi: https://doi.org/10.1101/2023.02.26.530159; this version posted February 27, 2023. The copyright holder for this preprint (which was not certified by peer review) is the author/funder, who has granted bioRxiv a license to display the preprint in perpetuity. It is made available under aCC-BY 4.0 International license.

Supplemental Figures:

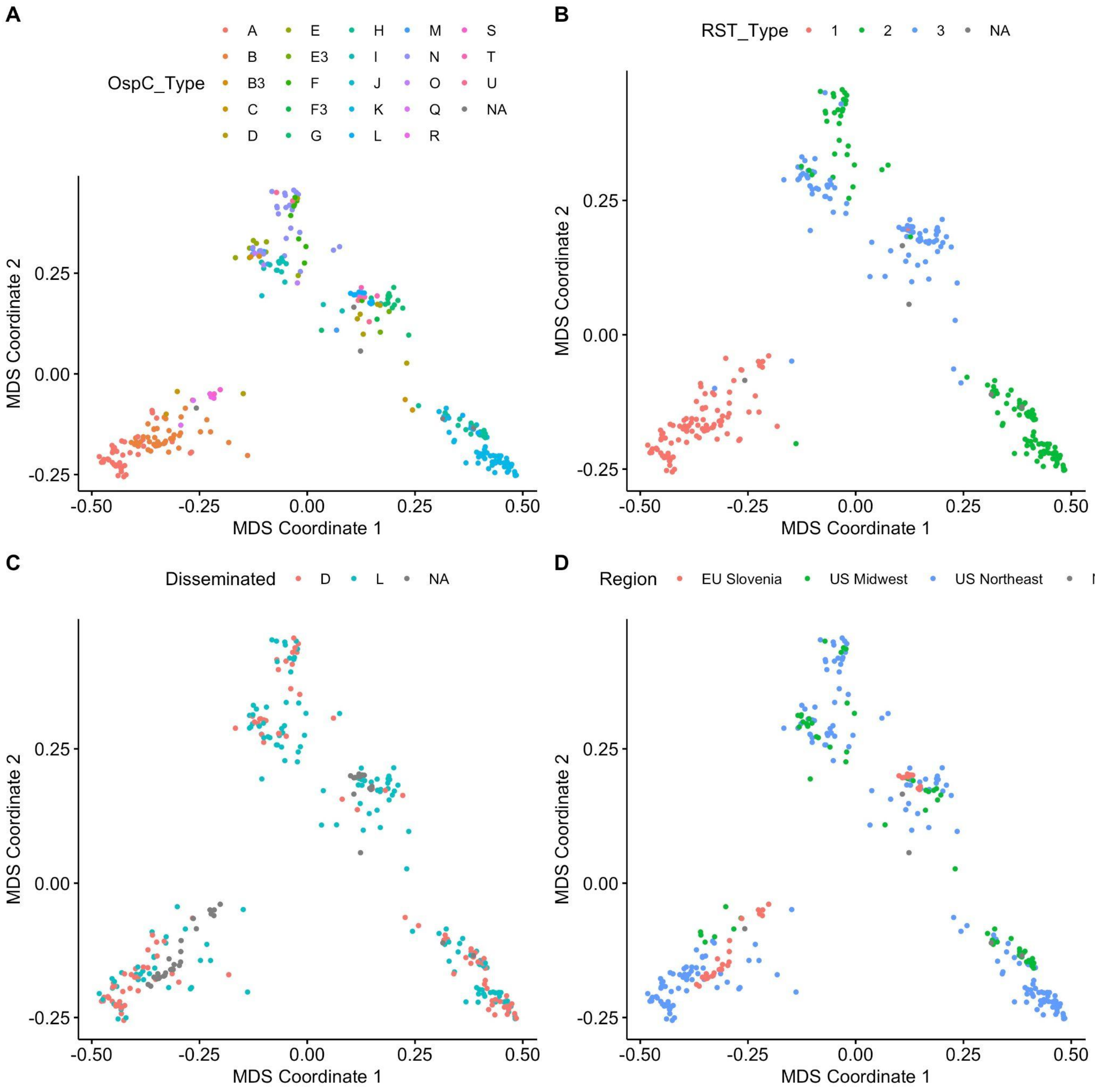

Supplemental Figure 1: Multidimensional scaling (MDS) reveals the population structure of US and Slovenian *Bbss* isolates.

bioRxiv preprint doi: https://doi.org/10.1101/2023.02.26.530159; this version posted February 27, 2023. The copyright holder for this preprint (which was not certified by peer review) is the author/funder, who has granted bioRxiv a license to display the preprint in perpetuity. It is made available under aCC-BY 4.0 International license.

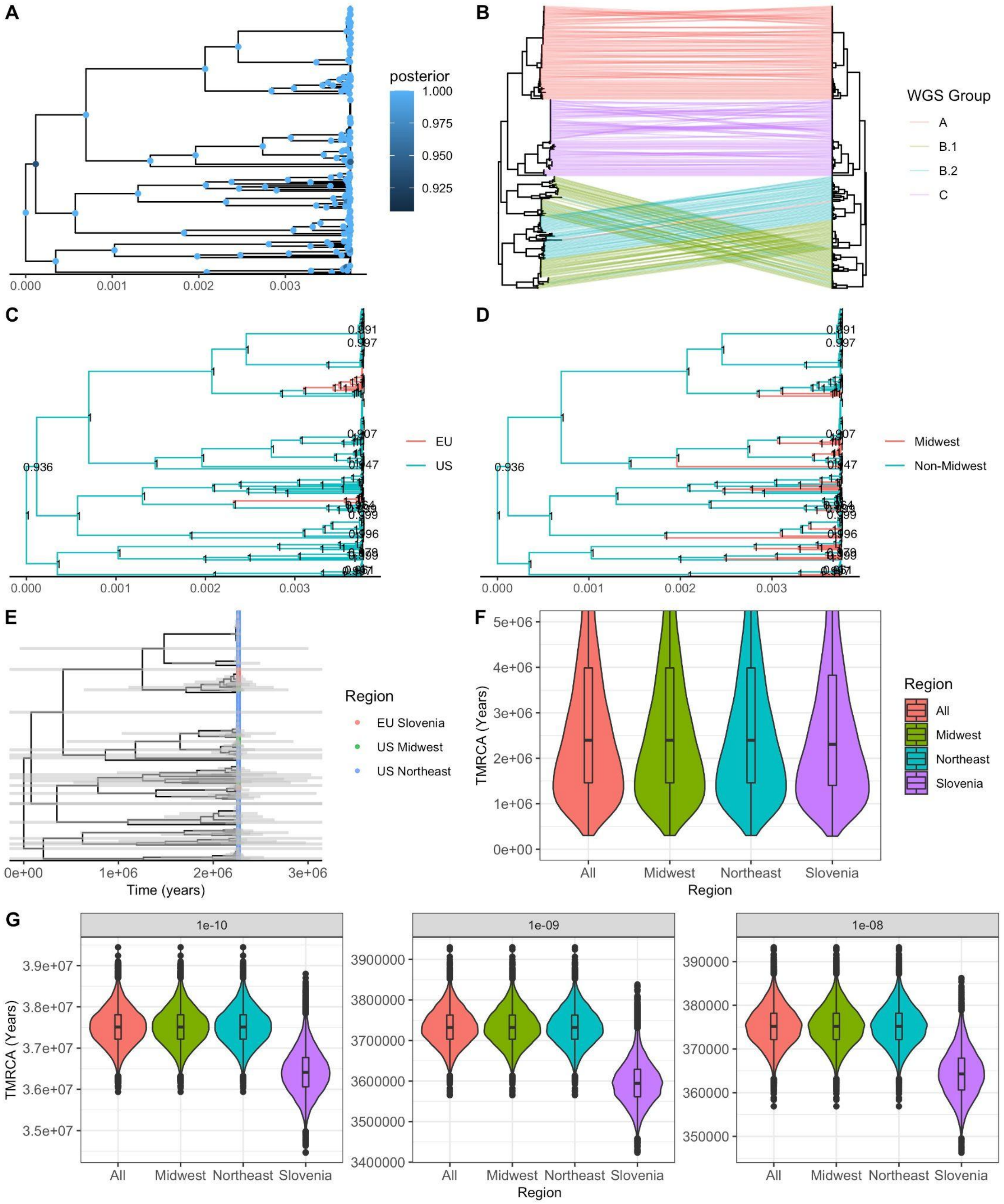

bioRxiv preprint doi: https://doi.org/10.1101/2023.02.26.530159; this version posted February 27, 2023. The copyright holder for this preprint (which was not certified by peer review) is the author/funder, who has granted bioRxiv a license to display the preprint in perpetuity. It is made available under aCC-BY 4.0 International license.

H

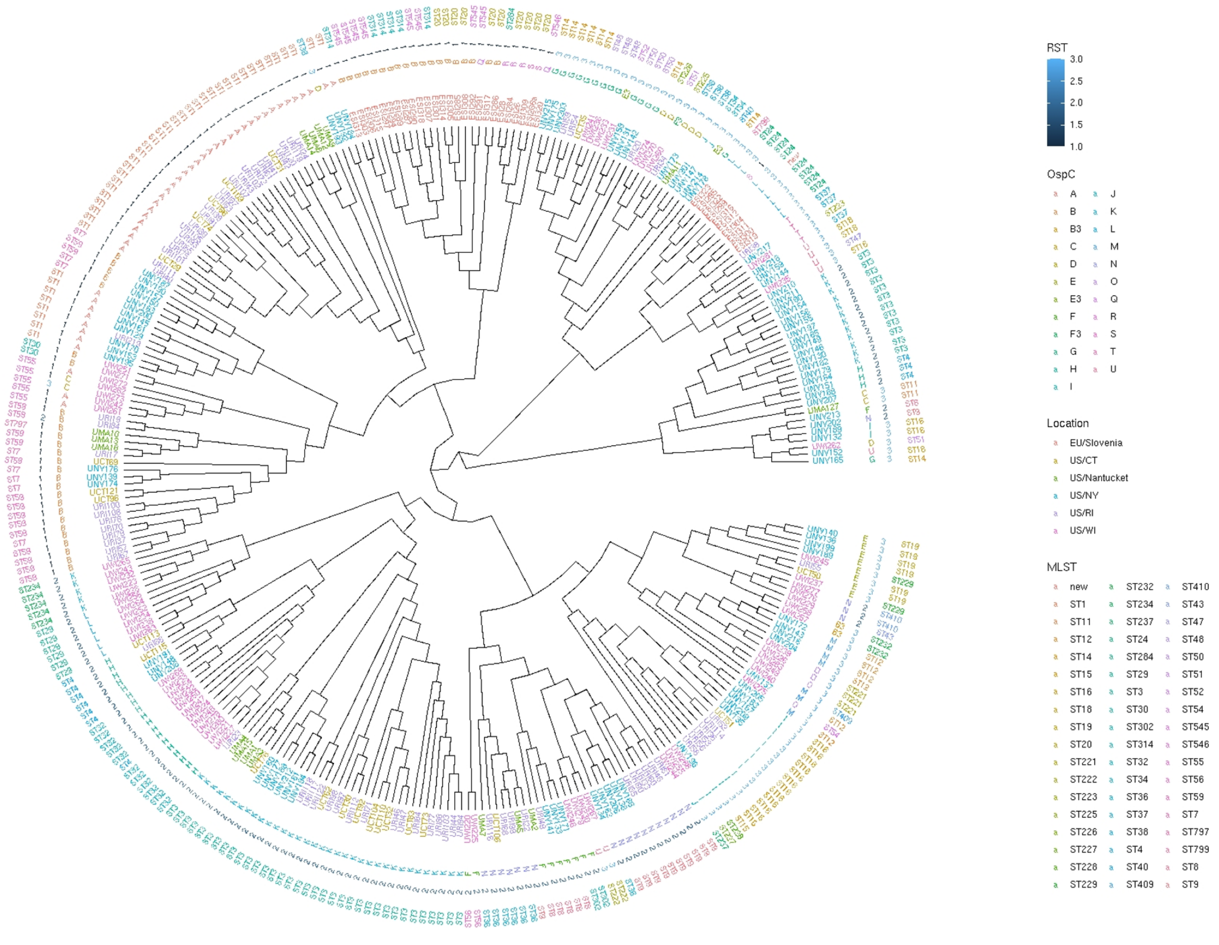

Supplemental Figure 2: A. Maximum clade credibility (MCC) tree. Nodes with posterior probability > 0.9 are colored. **B.** Maximum likelihood (left panel) and MCC tree, with identical tips connected with lines colored according to WGS group. **C.** MCC tree with nodes with posterior probability > 0.9 labeled. Tips from the US have been grouped and their most recent common ancestor are colored blue; all others are colored red. **D.** MCC tree with nodes with posterior probability > 0.9 labeled. Tips from outside the US Midwest have been grouped and their most recent common ancestor are colored blue; all others are colored red. **E.** Time-tree with 95% credible interval of node heights plotted as gray bars. **F.** Density of time to most recent common ancestry (TMRCA) for major subpopulations and the full sample set (root). An inset boxplot gives the median and IQR. **G.** Density of time to most recent common ancestry (TMRCA) for major subpopulations and the full sample set (root) under three different fixed-clock models with the clock rate set at $1x10^{-10}$ substitutions/site/yr (left panel), $1x10^{-9}$ substitutions/site/yr (middle panel), or $1x10^{-8}$ substitutions/site/yr (right panel). **H.** Core genome phylogeny of 299 whole-genome sequences. The phylogeny is shown as a cladeogram (branch length does not correspond to genetic distance). The tips are labeled with sample names. RST type, OspC type, location, and MLST type are annotated. Whole genome sequences recapitulates existing typing schemes while adding additional resolution. Geographic origin is associated with different branches of the tree. For example, Slovenian isolates cluster in two distinct branches.

bioRxiv preprint doi: https://doi.org/10.1101/2023.02.26.530159; this version posted February 27, 2023. The copyright holder for this preprint (which was not certified by peer review) is the author/funder, who has granted bioRxiv a license to display the preprint in perpetuity. It is made available under aCC-BY 4.0 International license.

bioRxiv preprint doi: https://doi.org/10.1101/2023.02.26.530159; this version posted February 27, 2023. The copyright holder for this preprint (which was not certified by peer review) is the author/funder, who has granted bioRxiv a license to display the preprint in perpetuity. It is made available under aCC-BY 4.0 International license.

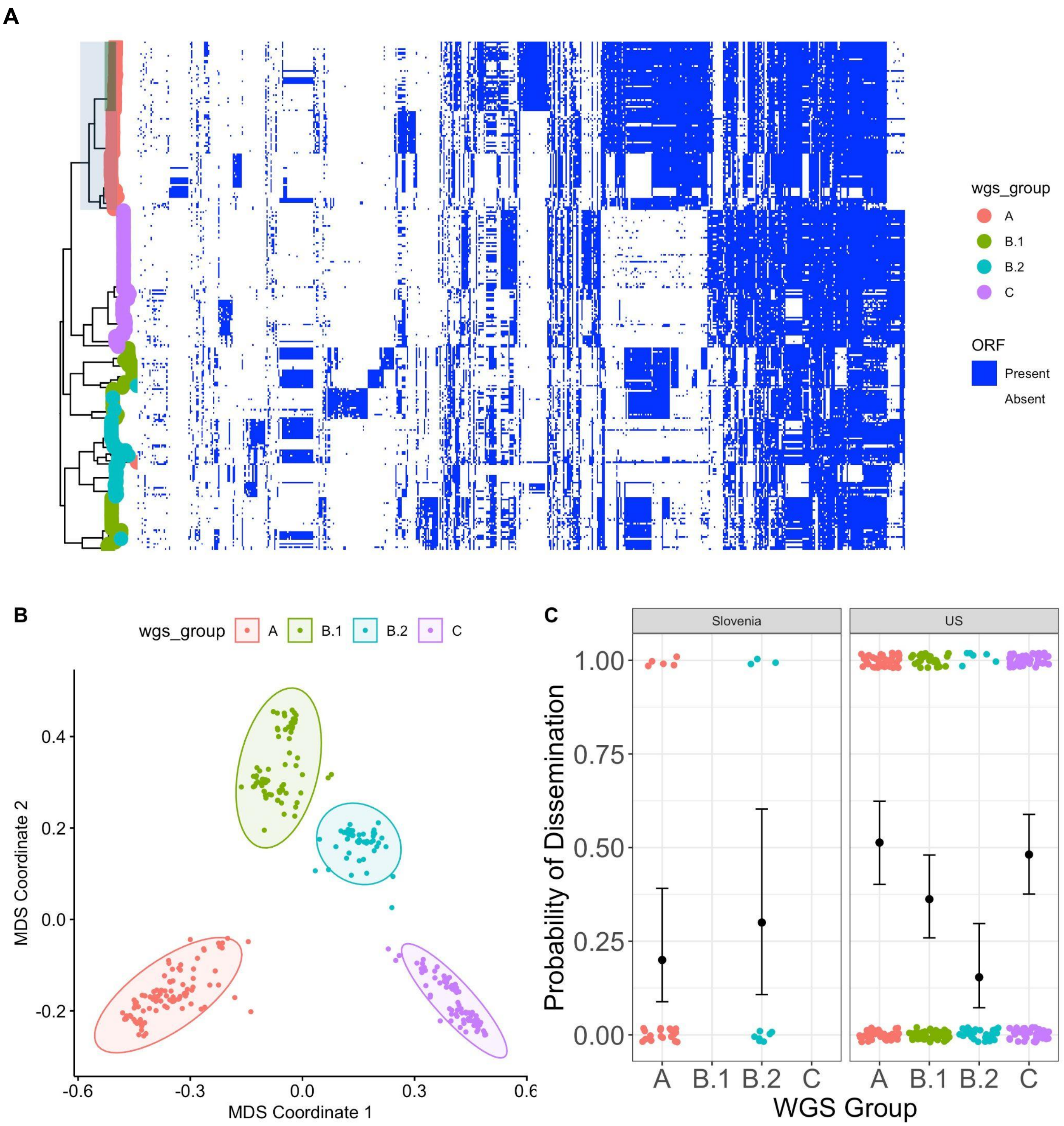

Supplemental Figure 3: A. Core genome phylogenetic tree colored by WGS groups A-C with group B divided into B.1 and B2; accessory genome presence/absence matrix is reproduced from Figure 5 to highlight accessory genome elements that correlate with B.1 and B.2 sublineages. The clade corresponding to RST1 is shaded in light blue and the clade corresponding to OspC type A is shaded in green. **B.** MDS plot with group B divided into B.1 and B.2. **C.** Probability of dissemination by genomic group using the four groups including B.1 and B.2.

bioRxiv preprint doi: https://doi.org/10.1101/2023.02.26.530159; this version posted February 27, 2023. The copyright holder for this preprint (which was not certified by peer review) is the author/funder, who has granted bioRxiv a license to display the preprint in perpetuity. It is made available under aCC-BY 4.0 International license.

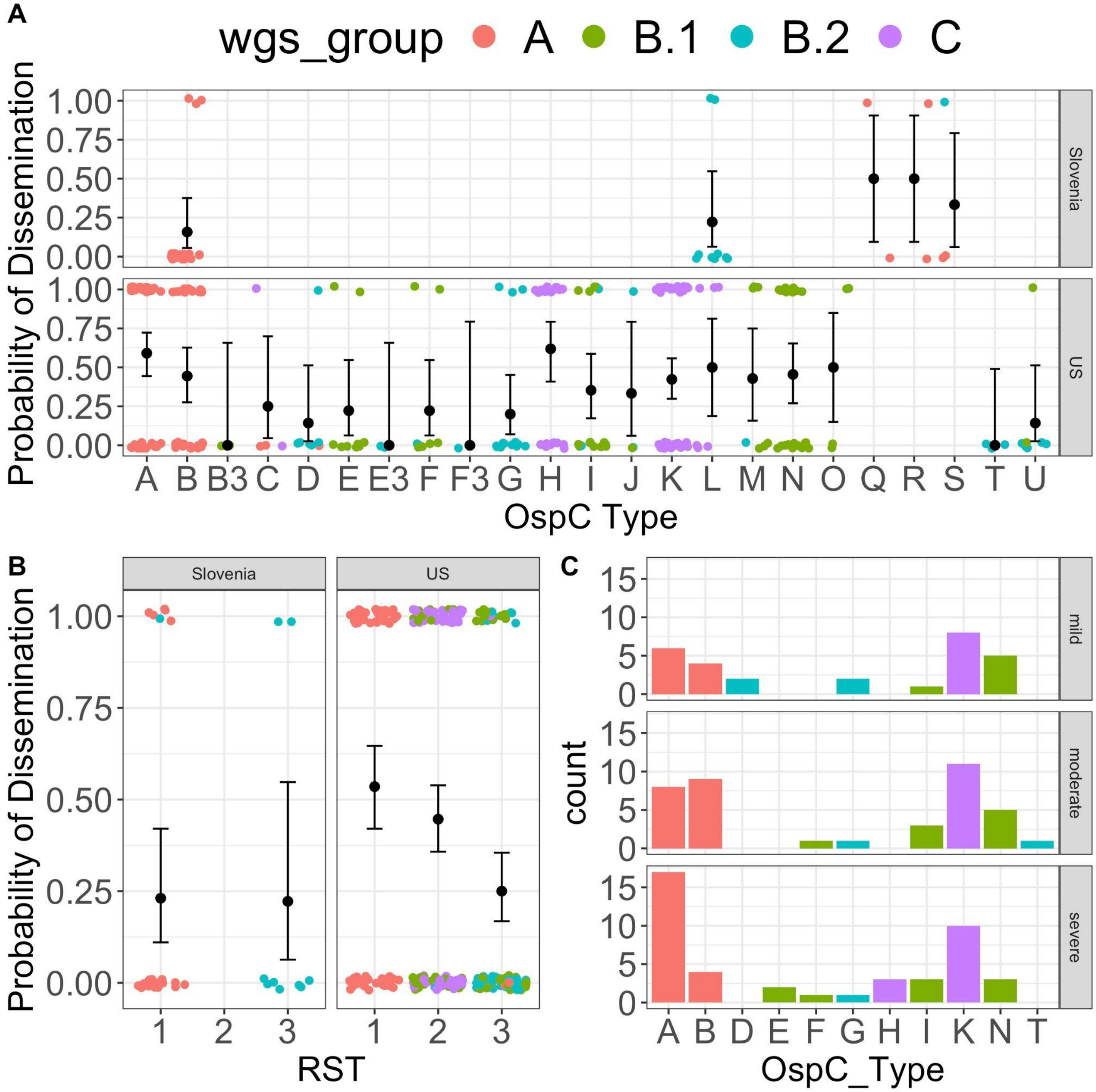

Supplemental Figure 4: Probability of dissemination by **(A)** OspC type and **(B)** RST. **C.** Severity of Lyme disease by OspC type with WGS group shown by color.

bioRxiv preprint doi: https://doi.org/10.1101/2023.02.26.530159; this version posted February 27, 2023. The copyright holder for this preprint (which was not certified by peer review) is the author/funder, who has granted bioRxiv a license to display the preprint in perpetuity. It is made available under aCC-BY 4.0 International license.

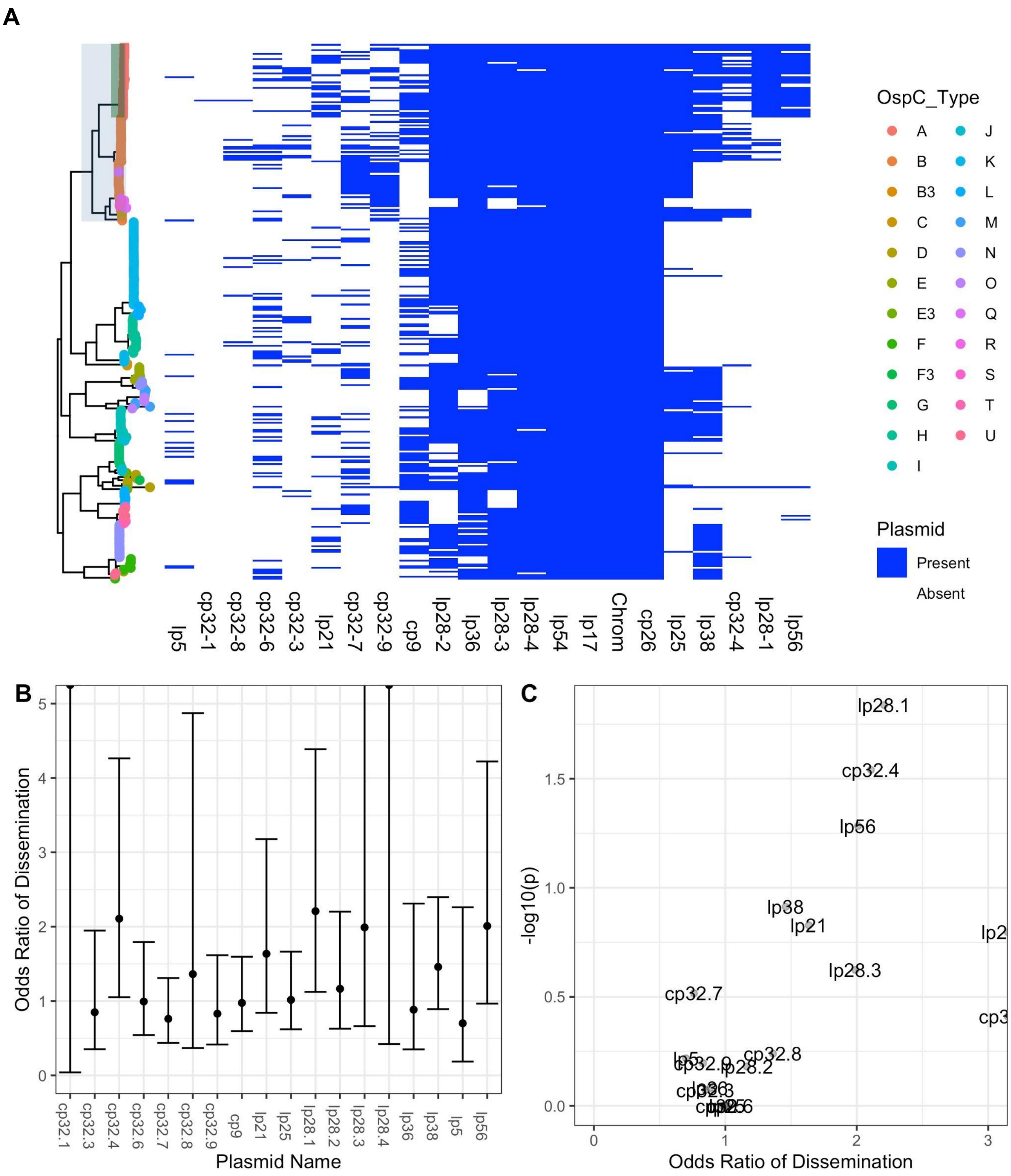

Supplemental Figure 5: A. Inferred presence / absence of a plasmid based on alignment of assembly contigs to the B31 reference. A plasmid is inferred as 'present' in the isolate if > 50% of the length is covered by aligned contigs in the de novo assembly for the genome of the corresponding isolate. The clade corresponding to RST1 is shaded in light blue and the clade corresponding to OspC type A is shaded in green. **B.** Odds ratio of dissemination and confidence interval by plasmid, inferred by Pfam32 sequences. **C.** Volcano plot displaying the

bioRxiv preprint doi: https://doi.org/10.1101/2023.02.26.530159; this version posted February 27, 2023. The copyright holder for this preprint (which was not certified by peer review) is the author/funder, who has granted bioRxiv a license to display the preprint in perpetuity. It is made available under aCC-BY 4.0 International license.

-log10 P value (as calculated using Fisher's exact test) and the odds ratio of dissemination for each plasmid, inferred by alignment of assembled contigs to the B31 reference sequence.

bioRxiv preprint doi: https://doi.org/10.1101/2023.02.26.530159; this version posted February 27, 2023. The copyright holder for this preprint (which was not certified by peer review) is the author/funder, who has granted bioRxiv a license to display the preprint in perpetuity. It is made available under aCC-BY 4.0 International license.

A

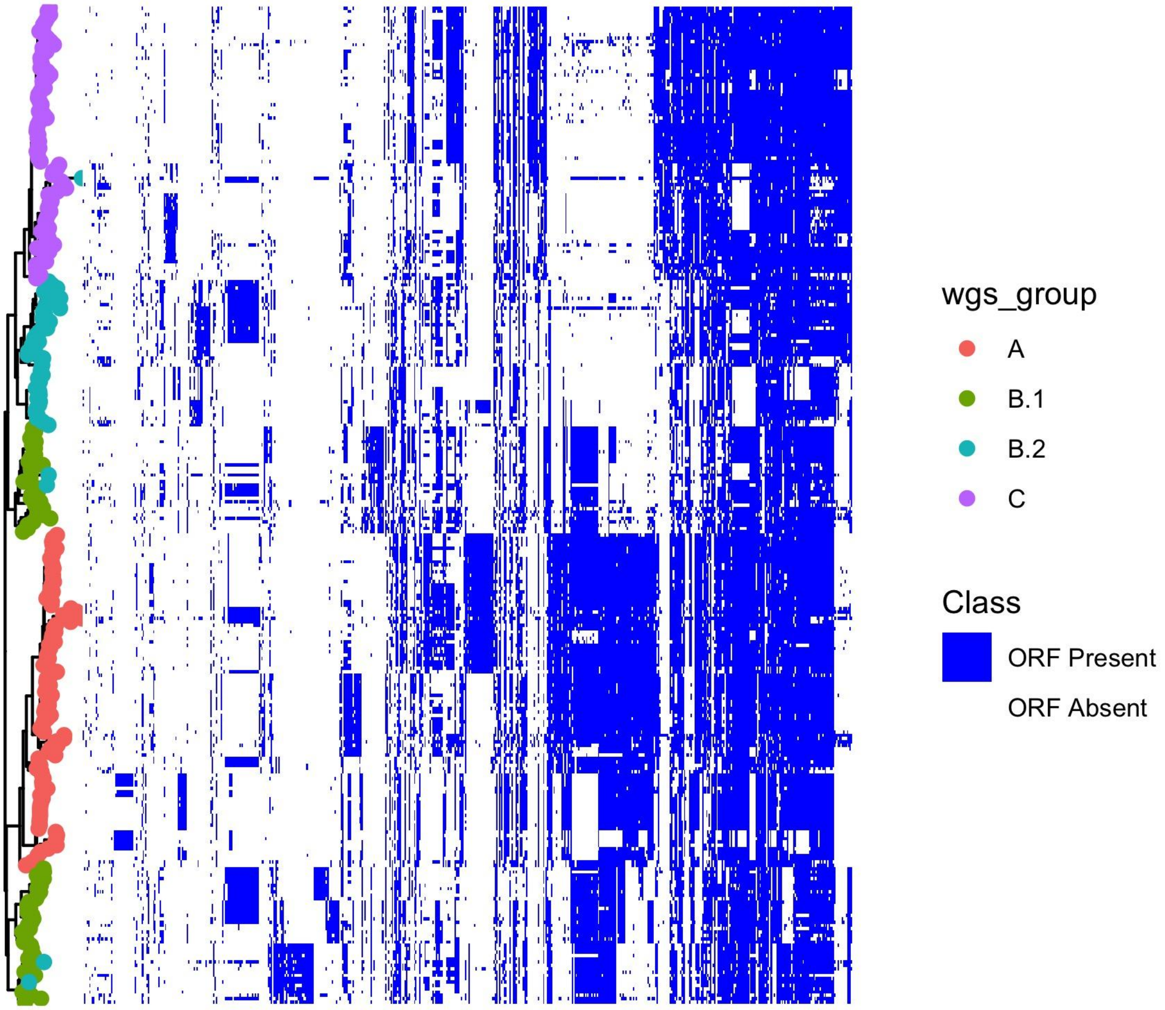

bioRxiv preprint doi: https://doi.org/10.1101/2023.02.26.530159; this version posted February 27, 2023. The copyright holder for this preprint (which was not certified by peer review) is the author/funder, who has granted bioRxiv a license to display the preprint in perpetuity. It is made available under aCC-BY 4.0 International license.

B

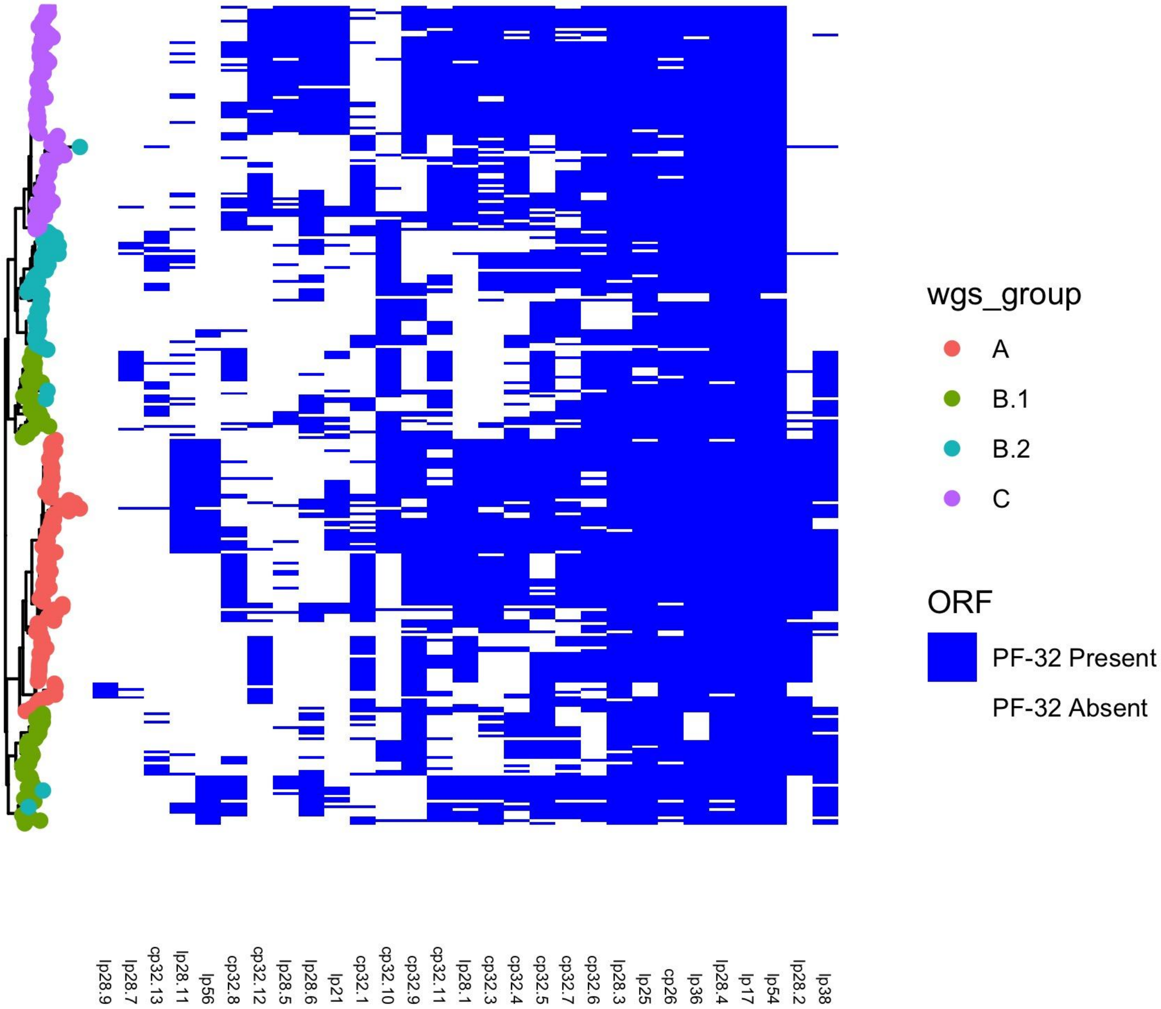

Supplemental Figure 6: A. Phylogenetic tree created from the accessory genome with accessory genome elements plotted according to their presence/absence in individual strains. **B.** Phylogenetic tree created from the accessory genome with PFam32 plasmid compatibility sequences plotted according to the presence/absence in individual strains.

bioRxiv preprint doi: https://doi.org/10.1101/2023.02.26.530159; this version posted February 27, 2023. The copyright holder for this preprint (which was not certified by peer review) is the author/funder, who has granted bioRxiv a license to display the preprint in perpetuity. It is made available under aCC-BY 4.0 International license.

A

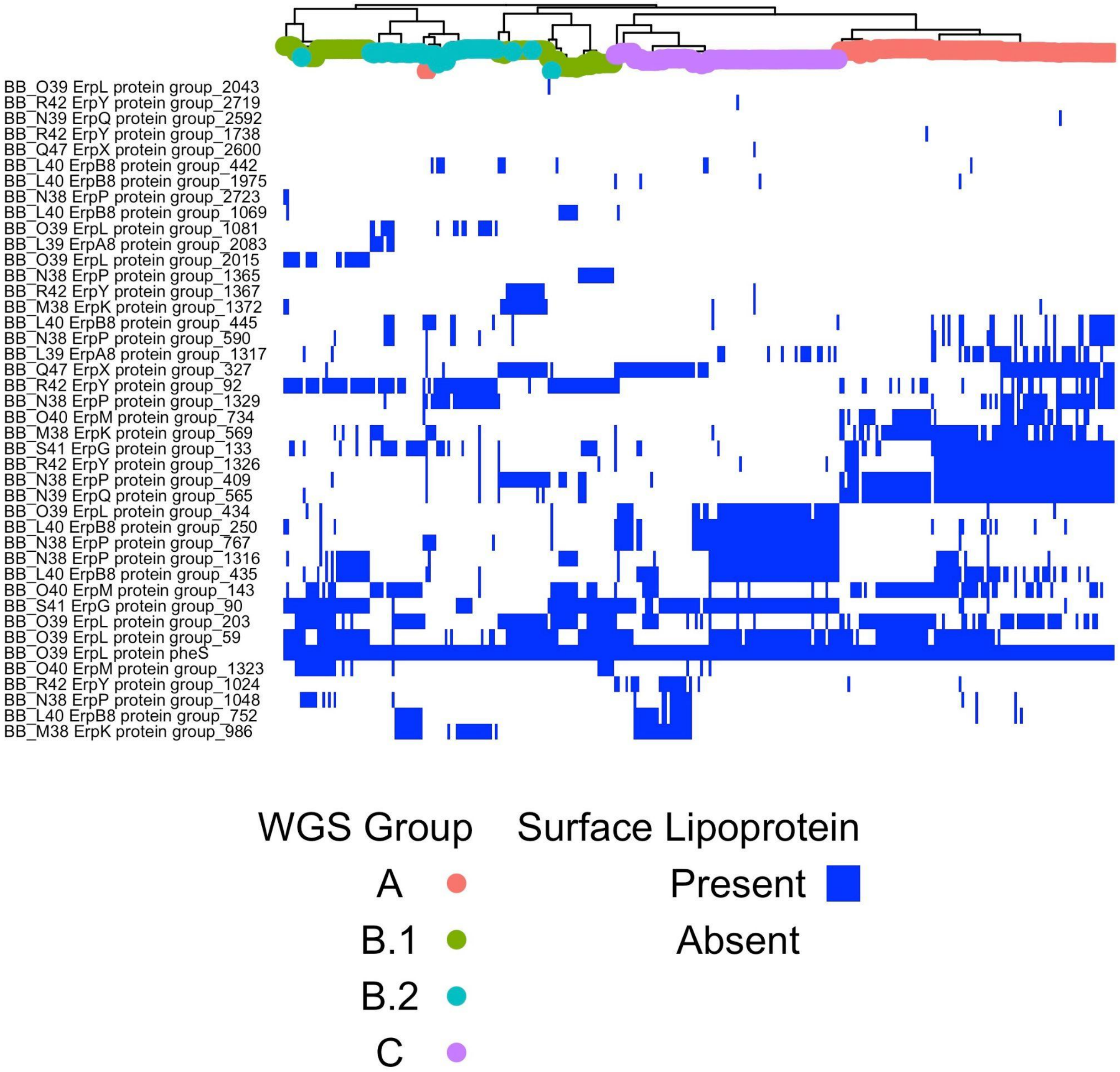

bioRxiv preprint doi: https://doi.org/10.1101/2023.02.26.530159; this version posted February 27, 2023. The copyright holder for this preprint (which was not certified by peer review) is the author/funder, who has granted bioRxiv a license to display the preprint in perpetuity. It is made available under aCC-BY 4.0 International license.

B

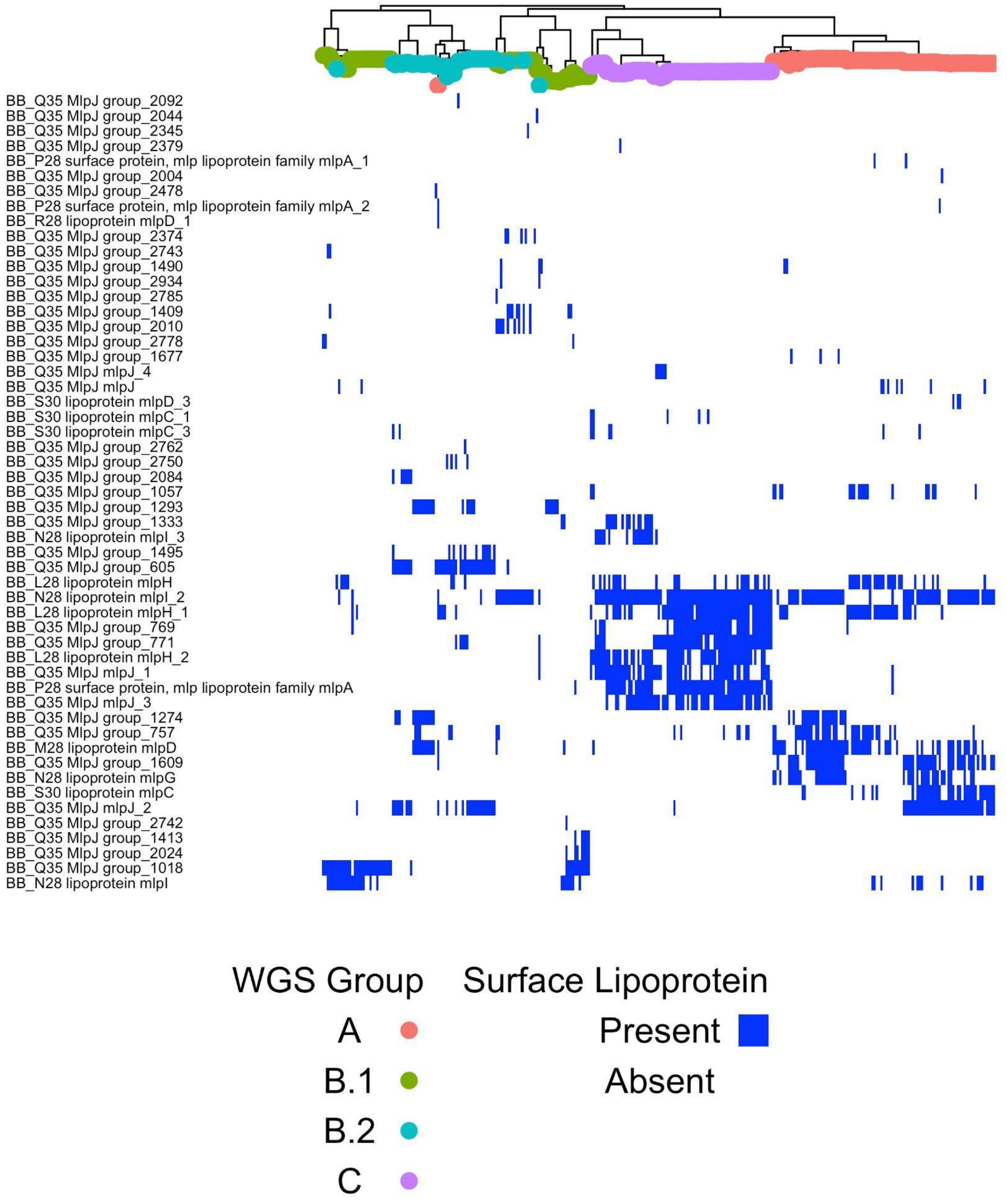

bioRxiv preprint doi: https://doi.org/10.1101/2023.02.26.530159; this version posted February 27, 2023. The copyright holder for this preprint (which was not certified by peer review) is the author/funder, who has granted bioRxiv a license to display the preprint in perpetuity. It is made available under aCC-BY 4.0 International license.

C

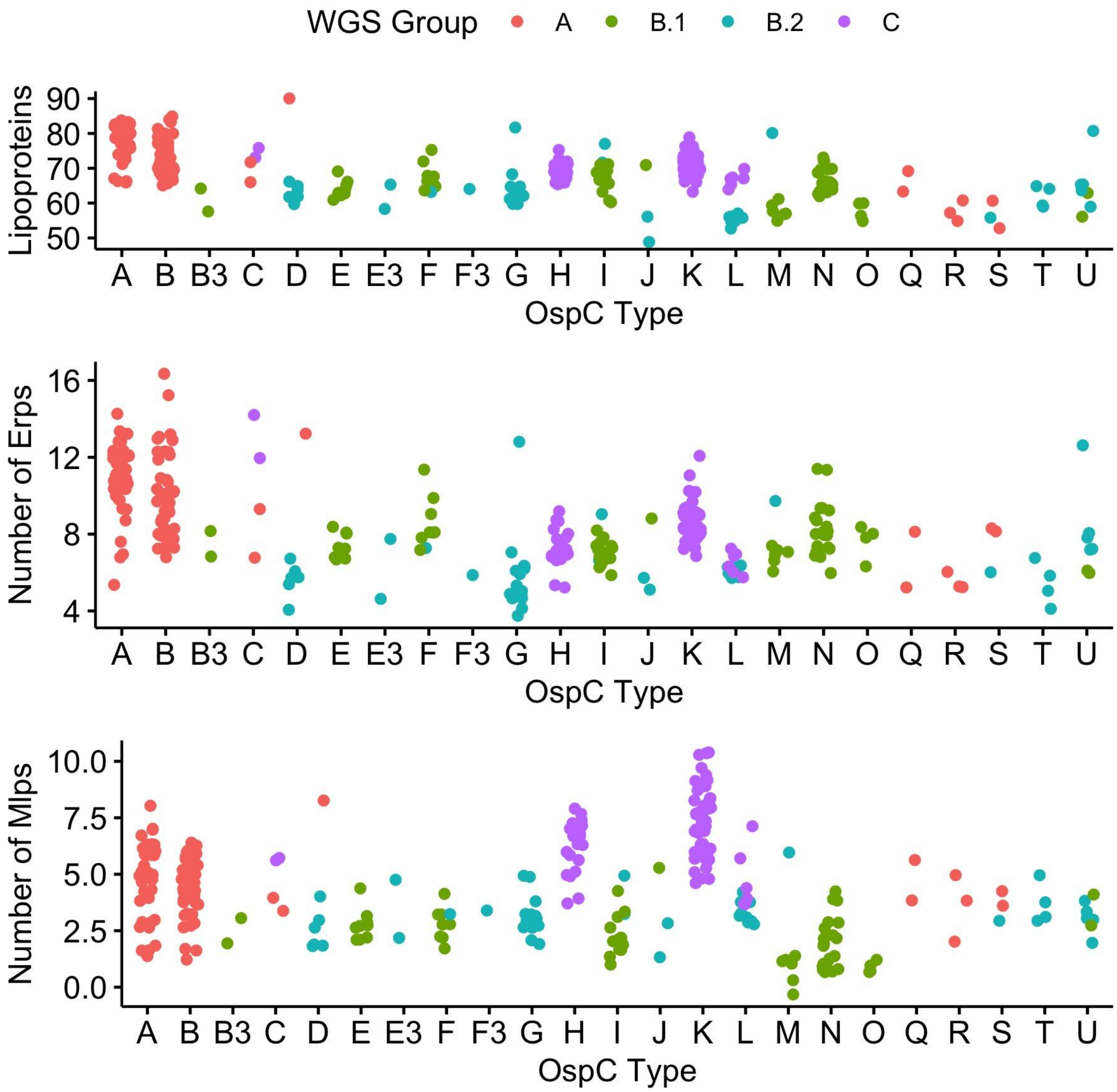

bioRxiv preprint doi: https://doi.org/10.1101/2023.02.26.530159; this version posted February 27, 2023. The copyright holder for this preprint (which was not certified by peer review) is the author/funder, who has granted bioRxiv a license to display the preprint in perpetuity. It is made available under aCC-BY 4.0 International license.

D

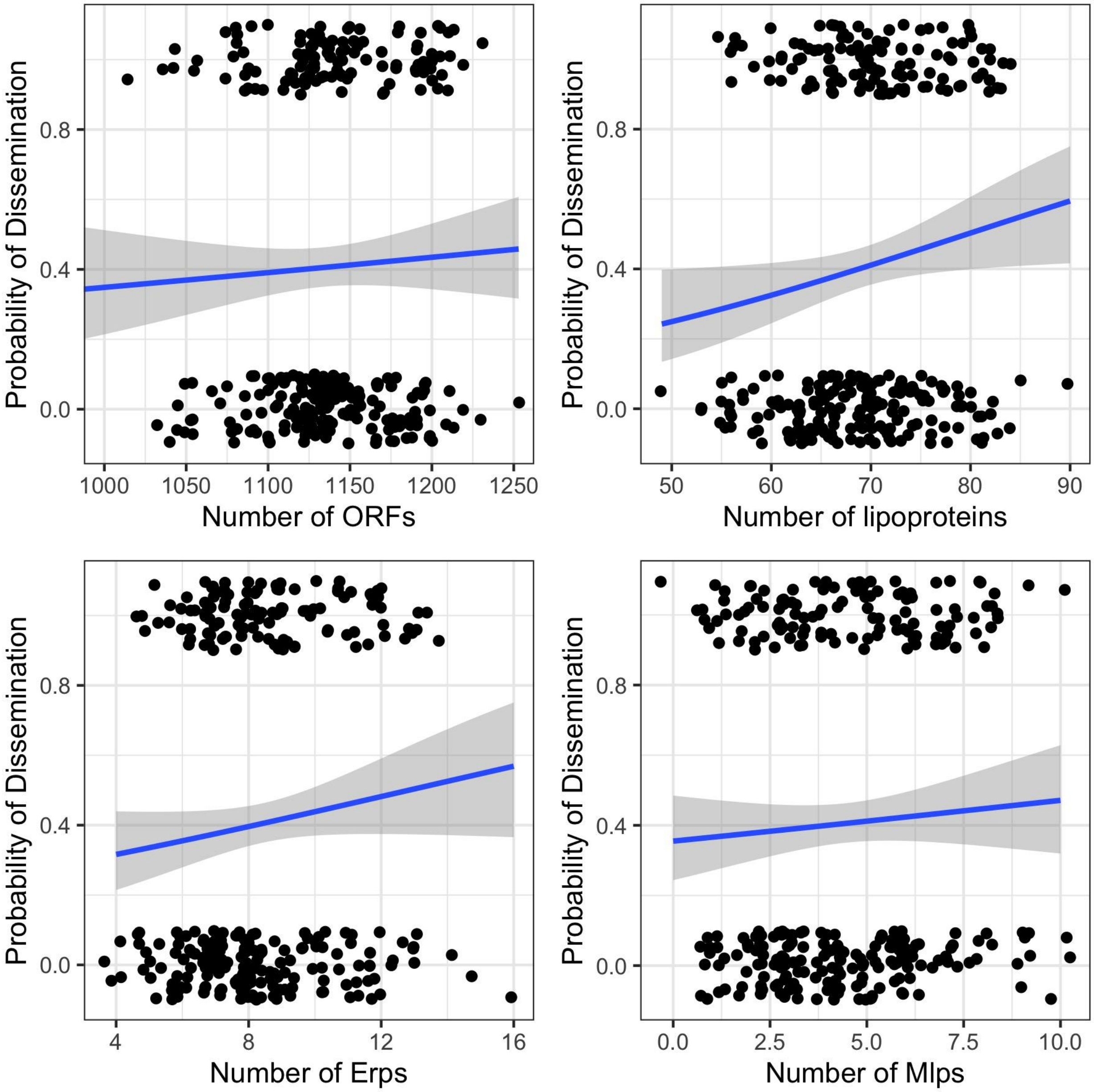

bioRxiv preprint doi: https://doi.org/10.1101/2023.02.26.530159; this version posted February 27, 2023. The copyright holder for this preprint (which was not certified by peer review) is the author/funder, who has granted bioRxiv a license to display the preprint in perpetuity. It is made available under aCC-BY 4.0 International license.

E

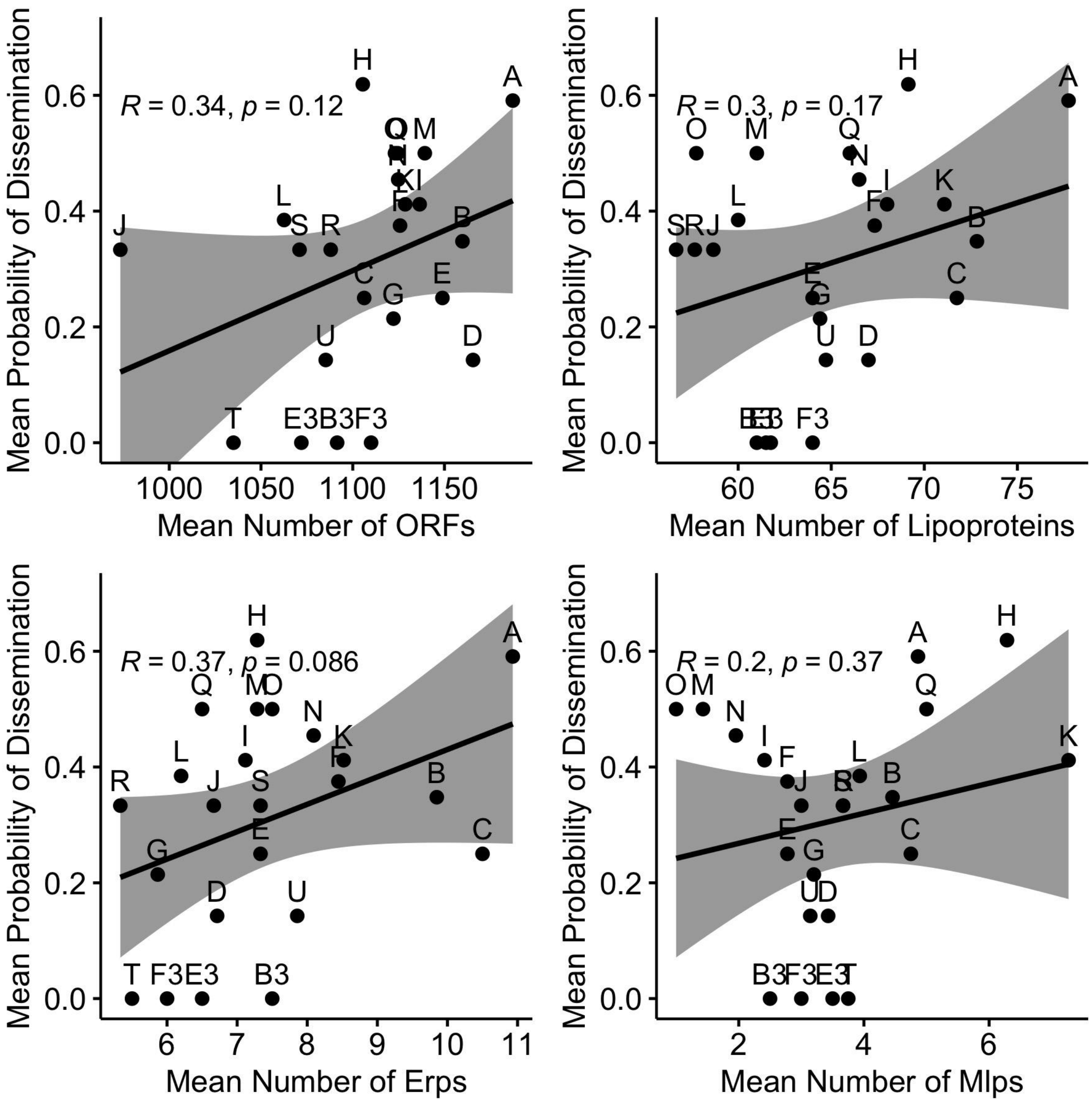

Supplemental Figure 7: A and **B.** Core genome phylogeny with presence/absence of Erp (C) orthologs and Mlp (D) orthologs. **C.** The number of surface-exposed lipoproteins (top panel), Erps (middle panel), and Mlps (bottom panel) by OspC type. **D.** Probability of dissemination by number of ORF (top left, logistic regression coefficient for slope, β_1 = 0.002 +/- 0.002, p = 0.450), number of surface-exposed lipoproteins (top right, β_1 = 0.037 +/- 0.017, p = 0.03, logistic regression), number of Erps (bottom left, β_1 = 0.087 +/- 0.053, p = 0.10, logistic regression), and number of Mlps (bottom right, β_1 = 0.048 +/- 0.055 p = 0.38, logistic regression). **E.** For each OspC type, mean probability of dissemination vs mean number of ORF (top left), mean number of surface-exposed lipoproteins (top right), mean number of Erps (bottom left), and mean number of Mlps (bottom right).

bioRxiv preprint doi: https://doi.org/10.1101/2023.02.26.530159; this version posted February 27, 2023. The copyright holder for this preprint (which was not certified by peer review) is the author/funder, who has granted bioRxiv a license to display the preprint in perpetuity. It is made available under aCC-BY 4.0 International license.

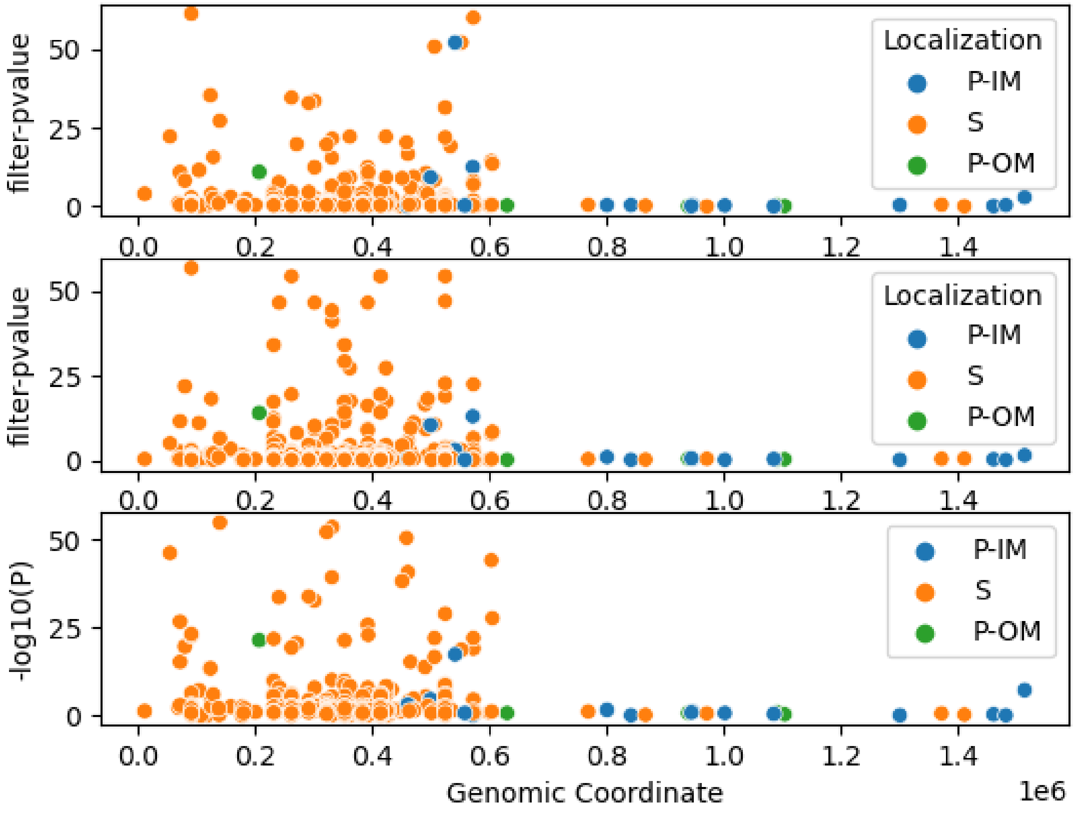

Supplemental Figure 8: Manhattan Plots showing the association of individual lipoproteins with OspC type A (top panel), Osp C type K (middle panel), and RST1 (bottom panel). Individual lipoproteins are annotated by their localization. P-IM: Periplasmic inner membrane. P-OM: Periplasmic outer membrane. S: surface.

bioRxiv preprint doi: https://doi.org/10.1101/2023.02.26.530159; this version posted February 27, 2023. The copyright holder for this preprint (which was not certified by peer review) is the author/funder, who has granted bioRxiv a license to display the preprint in perpetuity. It is made available under aCC-BY 4.0 International license.

List of supplemental data files

Supplemental Table 1: Summary table of isolates and phenotypes
Supplemental Table 2: List of isolates and phenotypes
Supplemental Table 3: Assembly statistics
Supplemental Table 4: Association statistics for plasmids, as inferred from PFam32 types.
Supplemental Table 5: Association statistics for plasmids, as inferred from B31 reference
Supplemental Table 5: Association statistics for lineage model
Supplemental Table 6: Association statistics for lineage model restricted to surface lipoproteins
Supplemental Table 7: Association statistics for OspC type A associations
Supplemental Table 8: Association statistics for OspC type K associations
Supplemental Table 9: Association statistics for RST1 associations
Supplemental Data File 1: List of ortholog groups with reference sequences
Supplemental Data File 2: High resolution version of presence/absence matrix in Figure 5B.

bioRxiv preprint doi: https://doi.org/10.1101/2023.02.26.530159; this version posted February 27, 2023. The copyright holder for this preprint (which was not certified by peer review) is the author/funder, who has granted bioRxiv a license to display the preprint in perpetuity. It is made available under aCC-BY 4.0 International license.

Supplemental Note 1:

The clock rate (in substitutions/site/year) for our initial model using a non-informative (CTMC rate reference) prior failed to converge—resulting in posterior 95% posterior density range from 5×10^{-25} substitutions/site/year to 1.2×10^{-8} substitutions/site/year—the implausibly small values at the lower end of the range are indicative of an insufficient temporal signal associated with genetic diversity in the core genome to establish an estimate without a priori assumptions. However, the inferred clock rate posterior had a clear single mode and a reasonable posterior mean (1.8×10^{-9} substitutions/site/year). To address this, we incorporated a priori information on mutation (gamma prior with shape 2, scale 1×10^{-9}, for which 95% of the density is between 3.55×10^{-10} substitutions/site/year and 4.47×10^{-9} substitutions/site/year, concordant with previous suggestions that the rate is approximately 1×10^{-9} substitutions/site/year[52]). This analysis suggests that the common ancestry of circulating human-infectious populations was remote (95% posterior density for Midwest strains: 380,000 - 11.8 million years; 95% posterior density for Slovenian strains: 379,000 - 11.5 million years; all strains: 380,000 years, 11.8 million years) (Figure 2E-F). We also ran models with a fixed rate across a variety of reasonable values (1e-10 to 1e-8) (Figure 2G).
